# Analyzing possible pitfalls of cross-frequency analysis

**DOI:** 10.1186/1471-2202-12-S1-P303

**Published:** 2011-07-18

**Authors:** Juhan Aru, Jaan Aru, Michael Wibral, Viola Priessemann, Wolf Singer, Raul Vicente

**Affiliations:** 1 Center for Research and Interdisciplinarity, Faculty of Medicine, Paris Descartes University, Paris, 75014 France; 2 Dept. Neurophysiology, Max-Planck Institute for Brain Research, Frankfurt am Main, 60528 Germany; 3 Frankfurt Institute for Advanced Studies, Frankfurt am Main, 60438 Germany; 4 MEG Unit, Brain Imaging Center, Goethe University, Frankfurt am Main, 60528 Germany; 5 Neural Systems and Coding, Max-Planck Institute for Brain Research, Frankfurt am Main, 60438 Germany

## 

One of the central questions in neuroscience is how neural activity is organized across different spatial and temporal scales. As larger populations oscillate and synchronize at lower frequencies and smaller ensembles are active at higher frequencies, a cross-frequency coupling would facilitate flexible coordination of neural activity simultaneously in time and space. Although various experiments have revealed amplitude-to-amplitude and phase-to-phase coupling, the most common and most celebrated result is that the phase of the lower frequency component modulates the amplitude of the higher frequency component. Over the recent 5 years the amount of experimental works finding such phase-amplitude coupling in LFP, ECoG, EEG and MEG has been tremendous (summarized in [1]). We suggest that although the mechanism of cross-frequency-coupling (CFC) is theoretically very tempting, the current analysis methods might overestimate any physiological CFC actually evident in the signals of LFP, ECoG, EEG and MEG. In particular, we point out three conceptual problems in assessing the components and their correlations of a time series. Although we focus on phase-amplitude coupling, most of our argument is relevant for any type of coupling.

**Figure 1:**
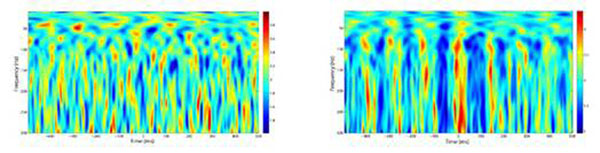
Time-evolution of the amplitudes of high frequency components locked to the trough of a low frequency component (from 4 to 8 Hz) for random noise from an atmospheric source (left) and its square (right).

*1*) The first conceptual problem is related to isolating physiological frequency components of the recorded signal. The key point is to notice that there are many different mathematical representations for a time series but the physical interpretation we make out of them is dependent on the choice of the components to be analyzed. In particular, when one isolates the components by Fourier-representation based filtering, it is the width of the filtering bands what defines what we consider as our components and how their power or group phase change in time. We will discuss clear cut examples where the interpretation of the existence of CFC depends on the width of the filtering process. *2*) A second problem deals with the origin of spectral correlations as detected by current cross-frequency analysis. It is known that non-stationarities are associated with spectral correlations in the Fourier space. Therefore, there are two possibilities regarding the interpretation of any observed CFC. One scenario is that basic neuronal mechanisms indeed generate an interaction across different time scales (or frequencies) resulting in processes with non-stationary features. The other and problematic possibility is that unspecific non-stationarities can also be associated with spectral correlations which in turn will be detected by cross frequency measures even if physiologically there is no causal interaction between the frequencies. *3*) We discuss on the role of non-linearities as generators of cross frequency interactions. As an example we performed a phase-amplitude coupling analysis of two nonlinearly related signals: atmospheric noise and the square of it (Figure 1) observing an enhancement of phase-amplitude coupling in the second signal while no pattern is observed in the first.

Finally, we discuss some minimal conditions need to be tested to solve some of the ambiguities here noted. In summary, we simply want to point out that finding a significant cross frequency pattern does not always have to imply that there indeed is physiological cross frequency interaction in the brain.
